# Solitary fibrous tumour: histological discoveries, behavioural aspects, risk assessment and therapeutical approaches

**DOI:** 10.1177/17588359251367320

**Published:** 2025-10-22

**Authors:** Claudia Di Prata, Paolo Del Fiore, Chiara Trevisiol, Saveria Tropea, Maria-Samaritana Buzzaccarini, Marco Krengli, Giovanni Scarzello, Ilaria Cosci, Marta Sbaraglia, Benedetta Chiusole, Fabio Murtas, Marcodomenico Mazza, Antonella Brunello, Marco Rastrelli, Simone Mocellin

**Affiliations:** Surgery Department, San Martino Hospital, Belluno, Italy; Soft-Tissue, Peritoneum and Melanoma Surgical Oncology Unit, Veneto Institute of Oncology, IOV-IRCCS, via Gattamelata, Padua 35128, Italy; Soft-Tissue, Peritoneum and Melanoma Surgical Oncology Unit, Veneto Institute of Oncology, IOV-IRCCS, Padua, Italy; Soft-Tissue, Peritoneum and Melanoma Surgical Oncology Unit, Veneto Institute of Oncology, IOV-IRCCS, Padua, Italy; Radiotherapy Unit, Veneto Institute of Oncology, IOV-IRCCS, Padua, Italy; Radiotherapy Unit, Veneto Institute of Oncology, IOV-IRCCS, Padua, Italy; Department of Surgery, Oncology and Gastroenterology, University of Padua, Padua, Italy; Radiotherapy Unit, Veneto Institute of Oncology, IOV-IRCCS, Padua, Italy; Department of Medicine, University of Padua, Padua, Italy; Department of Medicine, University of Padua, Padua, Italy; Department of Pathology, Azienda Ospedale-Università Padova, Padua, Italy; Oncology 1 Unit, Department of Oncology, Veneto Institute of Oncology, IOV-IRCCS, Padua, Italy; Oncology 1 Unit, Department of Oncology, Veneto Institute of Oncology, IOV-IRCCS, Padua, Italy; Soft-Tissue, Peritoneum and Melanoma Surgical Oncology Unit, Veneto Institute of Oncology, IOV-IRCCS, Padua, Italy; Oncology 1 Unit, Department of Oncology, Veneto Institute of Oncology, IOV-IRCCS, Padua, Italy; Soft-Tissue, Peritoneum and Melanoma Surgical Oncology Unit, Veneto Institute of Oncology, IOV-IRCCS, Padua, Italy; Department of Surgery, Oncology and Gastroenterology, University of Padua, Padua, Italy; Soft-Tissue, Peritoneum and Melanoma Surgical Oncology Unit, Veneto Institute of Oncology, IOV-IRCCS, Padua, Italy; Department of Surgery, Oncology and Gastroenterology, University of Padua, Padua, Italy

**Keywords:** antiangiogenetic agents, chemotherapy, radiotherapy, SFT, soft tissue sarcoma, solitary fibrous tumour, surgery

## Abstract

Solitary fibrous tumours (SFT) are fibroblastic mesenchymal tumours that can develop virtually at any site. It usually affects adults, and its incidence is estimated as 1 new case per million people per year. SFT is characterized by a gene fusion involving NAB2 (NGFI-A-binding protein 2) and STAT6 (signal transducer and activator of transcription 6); a higher nuclear STAT6 immunostaining can be seen in SFT cells, and it is considered an excellent marker for immunohistochemical diagnosis. The 2020 WHO classification defines two categories for SFT: intermediate (rarely metastasizing) and malignant. Many risk stratification models have been proposed to predict the behaviour of these tumour identities. In case of localized SFTs, the cornerstone of the treatment remains surgery with the aim of negative margins (R0) when technically feasible. In intermediate/high-risk SFTs with positive margins (R1/R2) with no possibility to re-resection or in meningeal high-risk SFTs, adjuvant radiotherapy (RT) could be performed. Neoadjuvant RT could also have a role in both extra-meningeal and meningeal localizations. Sole RT could have a role in managing non-resectable SFTs even in a curative intent. Medical therapy plays an important role in the metastatic/advanced SFTs. Conventional chemotherapy may be used, with doxorubicin and dacarbazine being active though yielding poor responses. Other active drugs with currently ongoing studies in SFT are eribulin and trabectedin. Antiangiogenetic treatments have also been shown to provide benefit in this histological subtype. An area of recent investigation is immunotherapy, with an ongoing randomized trial comparing nivolumab + ipilimumab versus pazopanib in advanced rare soft tissue sarcomas, including SFTs. Despite its rarity and therefore the difficulty in performing prospective randomized trials with a large number of patients, many promising results in perioperative (radiotherapy) or in the metastatic (medical therapy) setting have been obtained. This review overviews the main characteristics and provides the current knowledge on standard therapies.

## Introduction

Solitary fibrous tumours (SFT) are rare mesenchymal neoplasms that can develop virtually at any site (pleural, abdominal cavity, extremities, trunk, meningeal, and head and neck). It usually affects adults, and its incidence is estimated as 1 new case per million people per year.^1^ Its extreme rarity has made it extremely complex to understand its biological and clinical aspects. For a long time, it has been believed that SFTs are tumours with a quite indolent course and a relatively low metastatic potential. Due to the low number of cases, few studies have been carried on, but during the past years, some discoveries have been made, thus leading to a wider knowledge of this tumour and its behaviour, opening a route for new and promising approaches. The landmark studies regarding SFT are reviewed below, divided by subgroups (histological discoveries, behavioural aspects and risk assessments, therapeutical approaches) in a chronological order. Particular attention is given to different tumour sites, as many studies have been conducted based on tumour localization.

## Relevant sections

### Histological discoveries

In 2013, two different groups discovered a gene fusion between NAB2 (NGFI-A-binding protein 2) and STAT6 (signal transducer and activator of transcription 6) in SFTs.^2,3^ The NAB2-STAT6 gene fusion encodes a transcript that acts as a transcriptional activator, causing a high level of expression of the transcript itself. Subsequently, a higher nuclear STAT6 immunostaining can be seen in SFT cells, and it is considered an excellent marker for immunohistochemical diagnosis. This discovery made by Chmielecki et al.^2^ and by Robinson et al.^3^ created the basis for a better comprehension of the molecular history of the disease, underlying this gene fusion as a key marker in the development of SFTs. Since meningeal hemangiopericytoma and SFTs share the same gene fusion, the former has been removed as an entity because it is now well established that meningeal hemangiopericytomas are SFTs arising in the meninges and in newer classifications, the term ‘hemangiopericytoma’ has been removed.^4–6^

### Discoveries, behavioural aspects and risk assessment (metastatic or recurrence)

To predict the behaviour of these tumoural identities, in a study and in its subsequent revised work, Demicco et al.^7,8^ analysed various risk factors and proposed and refined a risk stratification model. In 2012, Demicco et al.^7^ identified 103 patients who underwent a resection of a primary extra-meningeal SFTs and noted that patient age, tumour size and mitotic index were the predictors of both metastasis and disease-specific mortality. In both cases, tumour size did not predict outcome. It was then developed a risk stratified model based on age (lower or higher than 55), tumour size (<5, 5–10, 10–15 and >15 cm) and mitotic figures (none, 1–3 and ⩾4), thus stratifying the risk of tumour aggressiveness into a low (no metastasis), intermediate or high one (at least 80% specificity for metastasis). While in their series of patients, the low-risk group had a zero chance to develop distant metastases or to die due to disease, the moderate and high-risk ones had, respectively, a 5-year metastasis-free rates of survival of 77% and 15%, a 10-year metastasis-free rates of survival of 64% and 0%, a 5-year disease-specific rates of survival of 93% and 60% and the 10-year disease-specific rates of survival of 93% and 0%. In 2017, Demicco et al.^8^ validated their previous work in an independent low-risk population of 79 primary extra-meningeal SFTs. Based on their previous risk stratification model, 57% of patients were at low risk, 29% at intermediate risk and 14% at high risk. In their previous work, they could not link necrosis as an independent risk factor for the development of metastasis due to a lack of adequate sample size for multivariate analysis, but they found that it was a significant risk factor in univariate analysis. They tested both sets of patients for a 4-variable model, including necrosis, and discovered that the distribution of risk percentage was quite different using this model: 66% of patients became low risk, 24% intermediate risk and 10% high risk. The Kaplan–Meier analysis showed a significant association between refined risk stratification and differences in metastatic risk. They also considered whether the mitotic count and necrosis calculated in the bioptic sample were similar to what was calculated in the resection sample and found out that in 30% of patients where risk was calculated on the bioptic sample, this leads to an underestimation of the real risk validated in the resection sample. The Demicco risk stratification model is designed to assess the risk of metastasis and not the local recurrence risk or the survival outcome. A recent Japanese study by Sugita et al.^9^ has evaluated the usefulness of a modified Demicco score that includes the Ki-67 index in place of mitotic activity. They found a significant difference in their series of 43 patients if they divided patients into two populations (low and intermediate/high risk), using both three-variable and four-variable models, both in the original Demicco score and in their modified Ki-67 score. Further studies with probably a larger series of patients could be conducted to verify these findings. Pasquali et al.^10^ retrospectively analysed 243 patients who underwent surgery for an extra-pleural or extra-meningeal primary SFT at 4 sarcoma centres in the period 2002–2011. For each patient, they gathered data on the patient, tumour and surgery characteristics. Recurrence was found in 18.5%, described as both locally (8.2%) and distant sites (10.3%). Hypercellularity, high mitotic rate and high levels of pleomorphism impacted disease-free survival (DFS) at univariate and multivariate analyses with a significant association, while necrosis was associated only in univariate analysis. They also found an inverse correlation between tumour size and DFS. No difference in DFS was found between primary and recurrence, while R1/R2 resection was associated with a higher risk of recurrence. Overall survival (OS) was significantly associated with high mitotic rate, necrosis, hypercellularity, pleomorphism, older age, deep-seated tumours and type of surgery (tumour excised with at least one organ or structure). OS was not associated with surgical margins, and tumour size was inversely correlated. No difference in OS was found between primary and recurrence. With these findings, they postulated a SFT recurrence score based on mitotic rate (low or high in case of more than 4× high-power field (HPF)), cellularity (low or moderate/high) and pleomorphism (low or moderate/high), obtaining a score divided in four groups: very low, low, intermediate or high risk of recurrence. Larger tumours that impacted inversely on DFS and OS were characterized by a lower incidence of adverse prognostic features (probably, tumour biology is somewhat more indolent if the tumour can grow to a large extent without spreading into distant organs). 1-, 3- and 5-year DFS based on this score was found to be 100% in all three cases for very low-risk SFTs, varying from 96.6% to 88.9% in low-risk SFTs, from 94.4% to 65.9% in intermediate-risk SFTs and from 78.5% to 52.6% (at both 3- and 5-year) for high-risk SFTs. Since this study evaluated the risk of local recurrence and very low- and low-risk patients showed no or very low risk of recurrence, they postulated a postoperative follow-up scheme that could allow a fairly wide follow-up interval for very low and low-risk patients, while intermediate- and high-risk patients could potentially benefit from a postoperative treatment (Table 1) Part A.

**Table 1.** Key landmark studies in risk assessment, surgery, radiotherapy and systemic treatment for SFT.

Evidence	Study design, data source, years include	Relevant study population	Key results
Part A: Evidence for risk assessment			
Demicco et al.^7^	Retrospective monocentric cohort (USA) 1986–2009	110	A risk stratification model based on age, size and mitotic index clearly delineated patients at high risk for poor outcomes. While small tumours with low mitotic rates are highly unlikely to metastasize, large tumours ⩾15 cm, which occur in patients ⩾55 years, with mitotic figures ⩾4/10 high-power fields, require close follow-up and have a high risk of both metastasis and death.
Demicco et al.^8^	Retrospective monocentric cohort (USA) 1996–2015	79	The findings confirmed the clinical utility of the previously published risk stratification model and support the inclusion of necrosis as a fourth variable in the model.
Sugita et al.^9^	Retrospective multicentric cohort (JAPAN)	43	Examine the prognostic usefulness of a modified version of the Demicco risk models that replaces the mitotic count with the Ki-67 labelling index. Modified risk models that include the Ki-67 labelling index are effective for the prediction of the prognosis in patients with SFT.
Pasquali et al.^10^	Retrospective multicentric cohort (UK-ITA-FRA) 2002–2011	243	Upon multivariable analysis, high mitotic rate (HR = 2.85, *p* = 0.002), cellular atypia (HR = 1.62, *p* = 0.015) and hypercellularity (HR = 1.82, *p* = 0.031) were significantly associated with recurrences. An SFT recurrence score has been provided to stratify the risk of recurrence.
Part B: Evidence for radiotherapy in SFT			
Haas et al.^13^	Retrospective multicentric cohort (NL-GER-CAN-UK-ITA-POL) 1990–2016	40	RT was applied with definitive intent in 16 patients and with palliative intent in 24. Clinically meaningful benefit for RT given with either definitive or palliative intent without surgery in SFT management
Haas et al.^14^	Retrospective multicentric cohort (NL-GER-CAN-UK-ITA-POL) 1990–2018	549	In patients with primary extrameningeal SFT, combining RT with surgery in the management is significantly associated with a reduced risk of local failures, especially in patients who have less favourable resection margins and in those who have tumours with a high mitotic count.
Lee et al.^16^	Retrospective multicentric cohort (KOR) 1995–2016	133	The findings support the role of postoperative RT in disease control of intracranial solitary fibrous tumor (SFT)/ hemangiopericytoma (HPC), irrespective of the surgical extent and grade. For LC, RT should enclose the tumour bed with a sufficient margin.
Part C: Evidence for systemic therapy in SFT			
Stacchiotti et al.^20^	Retrospective monocentric (ITA) 2012	35	Confirm sunitinib activity in SFT and long-lasting response.
Stacchiotti et al.^19^	Preclinical/clinical (ITA) 2014	6	In the dedifferentiated-SFT xenograft, pazopanib induced a marginal antitumour activity. When administered in patients, pazopanib showed a modest activity in terms of tumour growth stabilization, observed only in non-dedifferentiated cases.
Maruzzo et al.^23^	Prospective (UK) 2013	13	The findings confirm that the antiangiogenic drugs are active in SFT.
Martin-Broto et al.^24^	Single-arm, phase II trial, multicentric (SPA-ITA-FRA) 2014–2016	40	Confirm pazopanib option for systemic treatment of advanced malignant SFT, and provide a benchmark for future trials.
Stacchiotti et al.^21^	Phase II clinical study (ITA) 2015–2017	17	Confirm axitinib activity in progressive advanced SFT.
Stacchiotti et al.^22^	Phase II clinical study (ITA) 2019–ongoing	16	Testing eribulin activity in advanced SFT-ongoing.
Stacchiotti et al.^18^	Phase II clinical study (ITA) 2019–ongoing	50	Trabectedin vs adriamycin plus dacarbazine, in patients with advanced SFT (STRADA) ongoing.

HR, hazard ratio; LC, local control; SFT, solitary fibrous tumour; RT, radiotherapy.

In 2017, the French Sarcoma Group^11^ studied a series of 162 patients with extra-meningeal SFT from December 1975 to October 2014 to identify prognostic factors for OS, local recurrence and metastatic recurrence and to propose a risk calculator. For each patient, they gathered data on the patient, tumour and pathological characteristics. Median follow-up was 32.8 months, and local recurrence was observed in 12.3% of patients, while metastatic recurrence was observed in 16.7% of patients. 10-year OS was 76.8% and 20-year OS was 51.7% while 10- and 20-year Local recurrence interval (LRI) were 19.2% and 38.6%, respectively. 10- and 20-year MRI were 31.4% and 49.8%, respectively. Median OS was not reached, with 12.3% of patients deceased at the time of the evaluation, with 75% of tumour mortality. Prognostic factors of OS in univariate analysis were age (better outcome in younger than 60 yo patients) and mitotic count (worse outcome in high mitotic count). In multivariate analysis, age remained statistically significant while mitotic count tended to significance. Prognostic factors of LRI in univariate analysis were viscera localization and radiotherapy (RT), while in multivariate not only viscera localization and radiotherapy remained statistically significant, but also age. As for MRI, in univariate analysis, age, mitotic count and necrosis were statistically significant, while in multivariate analysis, mitotic count and tumour localization other than limb were significant. Based on these findings, they proposed a risk calculator for clinical practice where three prognostic groups were calculated for OS based on two unfavourable prognostic factors (age equal or greater than 60, mitotic count greater than 4), for LR were calculated four prognostic groups based on three factors (age less than 60, viscera localization, radiotherapy) and for metastatic recurrence four prognostic group based on three factor were calculated (age, mitotic count, tumour localization). According to the different prognostic groups, they calculated the cumulative incidence of Local Recurrence(LR) or Metastatic Recurrene (MR). This tool is useful in modulating the individualized approach tailored for each patient according to their risk of recurrence, even if the authors themselves suggest the need for validation with larger cohorts of patients.

More recently, another group^12^ published an integrated risk model that incorporates mitotic count, density of Ki-67+ cells and CD163+ cells and *MTOR* mutation. This model was proposed based on findings in 101 primary non-CNS patients, but the model itself was validated in both non-CNS and CNS SFTs. Zhang et al. found that all risk stratification models proposed before lacked optimal predictive accuracy and therefore studied the genomic landscape and the immune infiltrate of SFT to identify potential targets. They found a mutation of IDH1 that is present in 6.9% of soft tissue sarcoma (STS), and they also observed a high PD-L1 expression in 24.4% of SFT or immune cells. As for IDH1, they found a mutation (*IDH1* p.R132S) that is frequent in SFT patients considered malignant as per WHO classification, with high nuclear pleomorphism and cellularity. This mutation was not associated with progression-free survival (PFS), while alterations of *MTOR* were indeed associated with a worse PFS. The immune infiltrate was notably macrophages, with the high cell densities of CD68+ macrophages, CD163+ macrophages, and HLA-DPB1+ cells correlated with a shorter PFS, while high cell densities of CD20+B cells correlated with a longer PFS. CD3+ or CD8+ cell densities were not correlated with PFS. High PD-L1 expression was in both SFT and immune cells, but high PD-L1 expression in immune cells was associated with malignant classification, nuclear pleomorphism and tumour site. It was not associated with PFS. Based on these findings, the authors proposed a risk model with four variables: mitotic count, density of Ki-67+ and CD163+ and *MTOR* mutation. With this model, SFT patients were stratified into three groups as low risk, intermediate risk and high risk of recurrence (in terms of PFS). The stratification between intracranial versus extracranial SFTs is clinically valuable because these two subtypes differ significantly in terms of epidemiology, behaviour, treatment and prognosis. A structured comparison is shown in Table 2.

**Table 2.** Clinical stratification between intracranial versus extracranial SFTs.

Feature	Intracranial SFT	Extracranial SFT
Epidemiology	Rare, accounts for a small proportion of CNS mesenchymal tumours	More common, especially in the pleura, extremities, pelvis and retroperitoneum
Clinical presentation	Neurological symptoms (e.g. seizures, headache, cognitive deficits) depending on location	Often asymptomatic or presents as a painless mass; may cause compression symptoms
Histopathology	Similar histology (spindle cells, ‘patternless pattern’), though CNS-specific features may appear	Histologically similar, but certain markers (e.g. STAT6 nuclear staining) are consistent across locations
Behaviour and risk	Often more aggressive; higher recurrence and potential for CSF dissemination	Behaviour varies widely by site; it can range from benign to highly aggressive
Recurrence	Local recurrence is relatively common, even after gross total resection	Recurrence rates depend on the completeness of resection and histologic grade
Metastatic potential	Less frequent but possible, especially with anaplastic features	Known to metastasize, especially high-grade tumours (lungs, liver, bone)
Standard treatment	Maximal safe surgical resection + consideration of adjuvant radiotherapy	Wide local excision; adjuvant RT used selectively, especially for high-grade or incompletely excised tumours
Radiotherapy role	More frequently employed due to difficulty in achieving clean margins and higher recurrence risk	Considered in high-risk cases (positive margins, high mitotic index, recurrence)
Prognosis	Prognosis varies; recurrence-free survival is shorter, especially without ART	Generally favourable for low-grade tumours; poorer for malignant or dedifferentiated forms

RT, radiotherapy.

#### Clinical implications of stratification

Treatment planning: Intracranial SFTs often merit more aggressive adjuvant therapy due to the difficulty of achieving wide surgical margins.
Follow-up strategy: More intensive imaging surveillance is typically warranted for intracranial SFTs.
Risk assessment: While both types carry malignant potential, intracranial SFTs are more prone to early local recurrence, whereas extracranial SFTs may exhibit late metastasis, especially when arising from deep soft tissues.

### Prognostic markers in SFT

SFTs are rare mesenchymal neoplasms with heterogeneous clinical behaviour, ranging from indolent to highly aggressive forms. Accurate prognostication is essential for tailoring treatment intensity, follow-up strategies and informing patient counselling. Prognostic assessment in SFTs integrates histopathological features, molecular alterations, immunohistochemical profiles and – emerging more recently – exploratory circulating biomarkers.

#### Histopathological prognostic markers

Histology remains the primary source for risk stratification in SFT. Several morphological features have been consistently associated with adverse outcomes:

Mitotic activity: A mitotic count ⩾4 per 10 HPF is associated with increased risk of recurrence and distant metastasis.
Tumour size: Lesions ⩾10 cm in diameter correlate with poorer prognosis.
Tumour necrosis: The presence of necrosis suggests a higher degree of aggressiveness.
Cellularity and nuclear atypia: Increased cellular density and nuclear pleomorphism support a malignant phenotype.

These criteria are the basis for the Demicco risk model, which incorporates age, tumour size, mitotic rate and necrosis to stratify metastatic risk. This model has been validated across multiple cohorts and remains a practical tool in clinical decision-making.

#### Immunohistochemical and molecular prognostic markers

STAT6 and NAB2–STAT6 fusion: The NAB2–STAT6 fusion is pathognomonic of SFT and serves as a diagnostic hallmark rather than a prognostic indicator. Nevertheless, specific fusion variants may associate with distinct clinical behaviour, although more research is needed.
Ki-67 proliferation index: Elevated Ki-67 labelling correlates with more aggressive biology and may augment existing risk models when assessing proliferation potential.
p53 overexpression: Aberrant nuclear accumulation of p53 has been observed in a subset of aggressive or dedifferentiated SFTs, possibly reflecting underlying genomic instability.
ISG15 (interferon-stimulated gene 15): Recent studies suggest that high ISG15 expression is associated with poor OS in SFTs. ISG15 may contribute to drug resistance mechanisms and cancer stemness, making it a promising candidate for future therapeutic exploration.

#### Circulating and blood-based prognostic biomarkers

At present, no validated blood-based biomarkers are available for prognostication in SFTs. Routine serum tests (e.g. LDH, inflammatory markers) have not demonstrated consistent prognostic value. Investigational efforts into circulating tumour cells and circulating tumour DNA are ongoing, particularly in the context of dedifferentiated or metastatic disease, but clinical implementation is premature.

### Therapeutical approaches

The management of localized/oligometastatic STF has its cornerstone in a surgery aimed at wide resection. Considering that SFTs can potentially arise everywhere in the body, surgical approaches can be very different from one another, but the goal is to aim for a microscopically complete surgical resection, even if this could be really challenging (i.e. meningeal SFTs, pleural SFTs). The roles of RT and systemic therapies have been investigated with promising results.

#### Radiotherapy

One study evaluated the role of RT as a single treatment without surgical resection,^12^ a few studies^13,14^ with quite large series have been recently conducted, all of which are retrospective and typically analysing different sites (especially dividing intracranial, meningeal and extra-meningeal localization). Other studies^15^ have retrospectively collected data regarding the possible benefit and role of postoperative RT in the management of SFTs, with a somewhat smaller series of patients, adding their contribution in validating a possible role of RT, especially in an adjuvant setting. Haas et al.^13^ performed a retrospective study on 40 patients collected at 7 sarcoma centres who received RT without surgery for a locally advanced and/or metastatic SFT. Definitive intent of RT was applied in 16 patients and palliative one in 24 patients. Median follow-up was 62 months. Patients receiving definitive RT (approximately 60 Gy received) had an objective response rate of 67% with a 5-year local control (LC) rate of 81.3% and a 5-year OS rate of 87.5%. In the palliative settings, these rates were, respectively, 38% (with approximately 39 Gy received), 62.5% and 54.2%. Toxicities were mild with predominant grade I effects. In the curative intent group (16 patients), complete response was achieved in 20% of the patients, partial response (PR) in 47%, stable disease (SD) in 27% and progression disease (PD) in 7%. These findings suggest that RT could effectively be used in non-resectable or metastatic patients, even with a potentially curative intent, and also in palliative intent, it could be obtained a certain grade of benefit in terms of prolonged disease control. It is reasonable to assume that hypofractionation schemes, which are becoming increasingly widespread, may improve outcome, especially in the oligometastatic setting, in relatively radioresistant tumours such as sarcomas. Haas et al.^13^ performed a study on extra-meningeal SFTs from 7 different sarcoma centres, collecting data on 549 patients, with a median follow-up of 52 months. Patients who underwent surgery alone were 428 (78%), while patients who underwent surgery plus RT were 121 (22%). Localization of the tumours was in the extremities (32.4%), retroperitoneum (23.9%), pleura (19.9%), trunk (17.7%) and head and neck (6.2). Considering the two subgroups (S vs S + RT), the localization was divided into pleural versus retroperitoneal versus others. Regarding RT patients, 52% of the patients received it preoperatively and 48% postoperatively, but no significant difference was found comparing the two timings. A higher percentage of R1 resection was found in this group compared to surgery alone (35.9% vs 20.6%, *p*-value 0.004). No significant differences in local PFS were found in the two groups. Local progression was associated with retroperitoneal localization, higher mitotic count, R1/R2 margin status and bigger size. Overall, no differences were found between S and S + RT in local progression. When corrected for mitotic count and surgical margins, S + RT was significantly associated with a lower rate of local progression, but no significant OS differences were found. Haas et al.^13^ also performed a study on meningeal SFTs comparing surgery and surgery plus postoperative RT (PORT). This is a multicentric retrospective study on a small set of patients (*n* = 48) from seven sarcoma centres with meningeal primary localized SFTs. They showed that 15% of patients underwent surgery alone and 85% of patients underwent surgery and PORT. With a median follow-up of more than 5 years, they discovered that LC was better achieved in the group with the association of radiotherapy (5-year LC 90% vs 60% with surgery alone). They also found that R1 resection was associated with worse LC with a hazard ratio of 4.08. There was found no statistical difference between surgery alone and surgery plus RT in OS, while the most important univariate factor impacting OS was mitotic count. Since the relatively small numbers, the LC benefit of adding RT did not achieve a statistical significance, but the *p*-value arrived quite close, with a value of 0.052. Moreover, it is interesting to note that in this set of patients, none had a high risk of the Demicco score. Lee et al.^16^ performed a multi-institutional retrospective study (KROG18-11) on 133 patients with intracranial SFTs from eight centres who underwent a surgical (gross or subtotal) resection. In 64% of the patients, a PORT was performed. 10 year PFS and OS resulted in 34% and 80%, with a median follow-up of 76.9 months. In multivariate analysis, PORT reported a significant difference in LC rate and PFS, both with a *p*-value <0.001, with a benefit in PFS maintained in gross resection, in WHO grade II and subtotal resection. Gross resection and PORT groups showed the best outcome in 5-year PFS (89%) and LC (93%), while subtotal resection and without PORT showed the worst outcome with a 20% of 5-year PFS and 18% 5-year LC. PORT was not associated with improvement in OS, regional control or distant metastasis-free survival. In the PORT group, a margin of target volume of 0–0.5 cm showed a worse LC than a margin of 1.0–2.0 cm, even after adjusting for the local effect of surgical extent (*p* = 0.021) (Table 1 Part B). Recent advancements in machine learning (ML) have shown promise in creating predictive models for radiotherapy outcomes in SFTs. For instance, a study published in *World Neurosurgery* investigated the efficacy of ART in intracranial SFTs and applied ML techniques to develop accurate prognostic models.^17^ The researchers found that ML-based models could effectively predict patient survival outcomes, thereby assisting clinicians in making informed decisions regarding the use of ART in SFT cases (Table 3). The model utilized clinical and radiological features to stratify patients based on survival benefits from ART. Key findings included the following:

- Improved survival prediction when incorporating ML models compared to traditional statistical methods.
- Identification of patient subgroups that significantly benefit from ART, guiding individualized therapy.

For extracranial SFTs, ML models have been employed to predict tumour behaviour based on radiomics, histopathological markers and clinical variables. Studies utilizing MRI-based deep learning models have successfully differentiated aggressive from benign phenotypes, assisting in prognosis and treatment selection.

**Table 3.** ML-based models predicting patient survival outcomes and assisting in decisions regarding the use of ART in SFT.

Feature	Intracranial SFT	Extracranial SFT
Primary treatment	Surgery ± ART	Surgery ± ART (select cases)
Recurrence risk	Higher	Variable (depends on location)
ML utility	Predicting ART benefit, survival outcomes	Stratifying benign vs aggressive forms
Key ML features	Tumour radiomics, molecular markers, clinical history	MRI-based classification, Ki-67 prediction

ML, machine learning; SFT, solitary fibrous tumours.

ML-based predictive models enable precise stratification of intracranial versus extracranial SFTs, offering tailored radiotherapy recommendations. By leveraging advanced algorithms, these models enhance clinical decision-making, potentially optimizing patient survival and reducing unnecessary ART exposure.

#### Systemic therapy

In the locally advanced/metastatic settings, initial studies were focused on validating conventional chemotherapy utilized in STS into this subset, while subsequently, with new preclinical information, other typologies of therapies have been studied, particularly antiangiogenetic ones (Table 1 Part C).

##### Chemotherapy

Various retrospective and a few prospective studies have evaluated conventional chemotherapy, often adopted from metastatic STS settings, in patients with advanced SFTs. These studies investigated anthracycline-based combinations, dacarbazine monotherapy and trabectedin, consistently reporting limited efficacy in this population. Notably, the disease control achieved was often transient, with modest PFS and OS outcomes, particularly in non-dedifferentiated SFTs. Importantly, the first prospective phase II trial specifically designed for SFT stratified patients into different cohorts based on tumour subtype (formerly classified as ‘malignant/dedifferentiated’ and ‘typical’), all with advanced disease. This trial demonstrated that patients with typical SFTs displayed a more indolent course even in the metastatic setting. A critical observation was that antiangiogenic therapies yielded more than double the median PFS and OS compared to historical data on conventional chemotherapy, especially in the typical/malignant SFT cohort. Furthermore, patients achieving at least SD with an antiangiogenic agent were often maintained on sequential antiangiogenic therapies upon progression, resulting in survival figures that exceeded those historically observed with cytotoxic chemotherapy. These findings suggest that, in the absence of strong supporting data, the activity of conventional chemotherapy in advanced SFT should not be overemphasized, especially outside the dedifferentiated subtype. Two phase II studies are ongoing in this setting: STRADA (NCT03023124), a randomized phase II trial comparing trabectedin (1.3–1.5 mg/m^2^ every 3 weeks) versus doxorubicin + dacarbazine, and ERASING (NCT03840772), a single-arm phase II evaluating eribulin (ClinicalTrials.gov identifiers: NCT03840772 and NCT03023124). Preliminary STRADA data^17^ include 23 enrolled patients randomized to either arm A (trabectedin) or arm B (doxorubicin + dacarbazine). No objective responses per RECIST were observed in either group. SD was achieved in 45% (arm A) and 40% (arm B), with progressive disease in 5% and 10%, respectively. Patients were stratified into dedifferentiated, advanced typical and malignant SFT subgroups. Due to the absence of RECIST responses in the typical and malignant subtypes, enrolment was halted in those cohorts; recruitment continues only for dedifferentiated SFTs, although current numbers are insufficient for firm conclusions. Given these data, anthracycline-based chemotherapy remains a first-line option primarily due to historical practice and lack of superior alternatives in randomized settings. However, increasing evidence supports the preferential use of antiangiogenic agents – especially in non-dedifferentiated SFTs – where their efficacy may be superior in disease stabilization and survival. Second- and subsequent-line options may include ifosfamide, dacarbazine and trabectedin, although their benefit appears limited and not comparable to that of antiangiogenics in appropriate subtypes.

##### Antiangiogenetic agents

In the first decade of the 2000s, various studies were conducted that reported some activity of antiangiogenetic agents in SFTs. Based on these findings, a few prospective studies have been carried out particularly evaluating the role of pazopanib. Following preclinical and clinical studies of Stacchiotti et al.^18–22^ (ClinicalTrials.gov identifier: NCT03023124) and the single-centre prospective study of Maruzzo et al.^23^ A multicentre, single-arm, phase II trial by Martin-Broto et al.^24^ evaluated pazopanib (800 mg daily) in 36 adult patients with metastatic or unresectable malignant or dedifferentiated SFT, all of whom had progressed within the previous 6 months. Based on Choi criteria, 51% achieved PR, 26% had SD and 23% showed progression, with a median PFS of 5.6 months. By contrast, RECIST 1.1 criteria yielded lower response rates (6% PR, 60% SD, 34% PD). The 2-year OS was 73%, though median OS was not reached. These results position pazopanib as a viable treatment option for advanced malignant SFTs, particularly when assessed with Choi response criteria and also derived data in terms of overall response, OS and PFS.

##### Immunotherapy

Since some studies have been focused on the immune environment in SFT settings, immunotherapy is a promising approach. Currently, the role of PD-1/PD-L1 and, therefore, anti-PD-1 checkpoint inhibitors in STSs are under evaluation.

The SARC028 and AcSé Pembrolizumab trials^25,26^ collectively highlight the histology-dependent limitations of immune checkpoint inhibitors in rare sarcomas, including SFTs. While pembrolizumab has demonstrated activity in certain STS subtypes, its efficacy in SFTs remains limited. The SARC028 trial evaluated pembrolizumab in selected STS subtypes (undifferentiated pleomorphic sarcoma (UPS), liposarcoma, synovial sarcoma, leiomyosarcoma), but did not include SFTs. Notably, responses were observed primarily in UPS, underscoring histology-specific variability in immunotherapy responsiveness. The AcSé Pembrolizumab trial expanded the scope to include rare and ultra-rare sarcomas, enrolling four SFT patients. No objective responses were observed in this group, aligning with known immunological features of SFTs – low PD-L1 expression, low tumour mutational burden and limited immune infiltration. Collectively, these studies highlight the limited benefit of immune checkpoint inhibitors in SFTs, particularly in non-dedifferentiated variants. Future directions may involve combinatorial strategies (e.g. with antiangiogenics or radiation) to enhance immunogenicity in this typically ‘immune-cold’ tumour type.

## Discussion

SFTs are rare mesenchymal neoplasms, arising in every district of the human body and which were somehow considered to be a benign entity. Pivotal works that lead to the creation of risk scores of metastasis and local recurrence have helped specialists in managing these patients with reliable tools in predicting tumoural behaviour. Regarding therapeutical approaches, some important concepts apply. In case of localized SFTs, the cornerstone of the treatment remains surgery with the aim of negative margins (R0) when technically feasible. In intermediate-/high-risk SFTs with positive margins (R1/R2) with no possibility to re-resection or in meningeal high-risk SFTs, adjuvant RT could be performed. Neoadjuvant RT could also have a role in both extra-meningeal and meningeal localizations. Sole RT has also been studied with suggestions that it could have a role in managing non-surgically resected SFTs even in a curative intent. Re-irradiation (as re-resection) is possible in case of locally recurrent intracranial SFTs.

## Future directions

Systemic therapy plays an important role in the metastatic/advanced SFTs, though the activity of available drugs as of today is still poor. In this setting, conventional chemotherapy has shown limited activity, with antiangiogenetic drugs displaying interesting results. Another area of recent investigation is immunotherapy, with a phase III trial ongoing comparing nivolumab + ipilimumab versus pazopanib in advanced STSs, including SFTs (ClinicalTrials.gov identifier: NCT04741438). Overall, immunotherapy in SFTs remains investigational, with limited efficacy observed to date due to their immunologically ‘cold’ nature, low mutational burden and scarce PD-L1 expression, these features are further supported by the limited or absent expression of cancer/testis antigens such as NY-ESO-1 and MAGE-A4^27^ – antigens shown to be rarely expressed in SFTs compared to other high-grade sarcomas – suggesting that future benefit may depend on combinatorial strategies aimed at enhancing tumour immunogenicity. While current evidence confirms that SFTs are largely resistant to immune checkpoint blockade due to their cold immune microenvironment and low immunogenicity, this reinforces the need for innovative combination strategies – such as pairing immunotherapy with antiangiogenics or radiotherapy – to unlock meaningful clinical responses in selected high-risk or dedifferentiated cases.

So, in the last 10–15 years, many discoveries have been made, leading to a better comprehension of this entity, starting from its diagnosis and its risk assessments, both in terms of local and distant metastasis. Since there is a real difference in the behaviour of these tumours between low risk and high risk, further studies are required to keep investigating biological aspects that could lead to an even better comprehension of the pathology. Despite its rarity and therefore the difficulty in performing prospective randomized trials with a large number of patients, many promising results in perioperative (RT) or in the metastatic (medical therapy) setting have been obtained. Further studies are needed to improve our knowledge. In summary, this review offers an integrated and updated overview of SFTs, combining recent histological insights, behavioural patterns and risk stratification – particularly through the clinical distinction between intracranial and extracranial SFTs. It also highlights emerging therapeutic perspectives, including the evolving role of radiotherapy, the limited efficacy of chemotherapy in non-dedifferentiated subtypes and the increasing value of antiangiogenic agents. Notably, the inclusion of ML-based predictive models for outcome estimation and decision-making represents a novel element, bridging traditional clinicopathological approaches with precision oncology.

## Declarations

**Ethics approval and consent to participate:** Not applicable.

**Consent for publication:** Not applicable.

**Availability of data and materials:** Not applicable.
